# Experimental Research on the Dam-Break Mechanisms of the Jiadanwan Landslide Dam Triggered by the Wenchuan Earthquake in China

**DOI:** 10.1155/2013/272363

**Published:** 2013-06-04

**Authors:** Fu-gang Xu, Xing-guo Yang, Jia-wen Zhou, Ming-hui Hao

**Affiliations:** ^1^State Key Laboratory of Hydraulics and Mountain River Engineering, Sichuan University, Chengdu, Sichuan 610065, China; ^2^State Key Laboratory of Geohazard Prevention and Geoenvironment Protection, Chengdu University of Technology, Chengdu, Sichuan 610059, China; ^3^College of Water Resources & Hydropower, Sichuan University, Chengdu, Sichuan 610065, China

## Abstract

Dam breaks of landslide dams are always accompanied by large numbers of casualties, a large loss of property, and negative influences on the downstream ecology and environment. This study uses the Jiadanwan landslide dam, created by the Wenchuan earthquake, as a case study example. Several laboratory experiments are carried out to analyse the dam-break mechanism of the landslide dam. The different factors that impact the dam-break process include upstream flow, the boulder effect, dam size, and channel discharge. The development of the discharge channel and the failure of the landslide dam are monitored by digital video and still cameras. Experimental results show that the upstream inflow and the dam size are the main factors that impact the dam-break process. An excavated discharge channel, especially a trapezoidal discharge channel, has a positive effect on reducing peak flow. The depth of the discharge channel also has a significant impact on the dam-break process. The experimental results are significant for landslide dam management and flood disaster prevention and mitigation.

## 1. Introduction

Landslide dams are formed rapidly (minutes to hours) by geological hazards, such as landslides [1, 2]. Because the material that makes up a landslide dam comes mainly from the surrounding mountain and is not artificially stabilised, it is much looser, less compact, and has lower cohesive strength than artificial dams which makes it more susceptible to burst failure [3, 4]. Dam failure disasters generally result in a huge loss of life and the destruction of both property and the natural environment [5]. From 1954 to 2006, there were 3498 dam failures in China, 90% of which were earth dams, with homogeneous earth dams accounting for 85% of all failures [6]. Earthquakes are the main cause of landslide dam formation; for example, the Wenchuan earthquake formed 256 landslide dams [7].

Numerous researchers have investigated landslide dam breaks using laboratory experiments and numerical simulations [8]. Miller and Chaudhry (1989) recorded water levels of dam break flows in a channel composed of two straight segments connected by a 180° bend [9]. Bellos et al. (1992) conducted series of experiments in a converging-diverging flume with both dry and wet beds [10]. The positive and negative wave fronts of dam breaks were investigated experimentally in a horizontal, smooth, and rectangular channel with a dry bed [11]. Soares Frazão and Zech (2002) obtained water levels and the velocity distributions in a channel with a sharp 90° bend [12]. There is little research on the influence of the discharge channel on dam breaks. There are many numerical studies concerning dam break flows for irregular downstream channels [13–15]. Mohapatra and Bhallamudi (1996) numerically investigated the effects of contractions and expansions on dam break flow depth at the dam site [16]. Aureli et al. (2000) studied dam break flow involving shock formation due to channel contraction using both experiments and numerical simulations [17]. However, the composition and failure mechanisms of landslide dams are very complicated, and it is difficult to truly capture all aspects of the dam break of a landslide dam using only numerical methods. 

The failure of landslide dam is very complicated process, and understanding dam failure mechanisms requires additional, multidisciplinary research [6]. Research of dam break mechanisms is very significant for understanding dam failures, dam development processes, disposal dam breaks, and sudden flood disasters, as well as formulating emergency action plans. This study uses the Jiadanwan landslide dam for several experiments designed to study failure mechanisms of a landslide dam under varying conditions, thereby significantly increasing our understanding of landslide dam breaks. 

## 2. Experimental Method and Program

### 2.1. The Jiadanwan Landslide Dam

**Figure 1:**
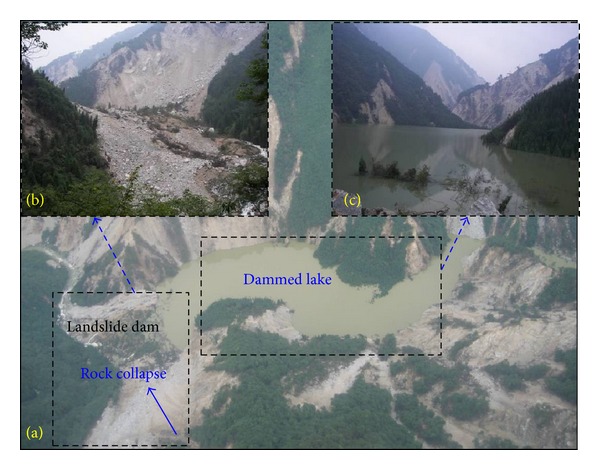
The Jiadanwan landslide dammed lake, (a) aerial photo, and (b) site photo of the landslide dam; (c) site photo of the dammed lake.

The Jiadanwan landslide lake is located near Dujiangyan city, Sichuan province, China. The river valley has an asymmetric “V” shape, with one wall steeper than the other. The Wenchuan earthquake caused a rock mass in the left bank slope to slide nearly 200 m down to the right bank. These landslide deposits blocked the Baisha River and formed a landslide dam. The resulting dammed lake has a capacity of approximately 6.1 × 10^6^ m^3^. The dam is *≈*200 m long, *≈*160 m wide, and *≈*60 m high (as shown in Figure 1). 

The dam is composed of an aggregate of granite boulders and soil, with rock grain sizes ranging from 0.04 to 0.80 m. Most of the dam material has a size of less than 0.40 m, and *≈*12% of the dam is made of soil. There is an artificial discharge channel on the left side of the landslide dam. If the Jiadanwan landslide dam were to break, the dam break flood would cause enormous downstream damage. 

### 2.2. Experimental Material

**Figure 2:**
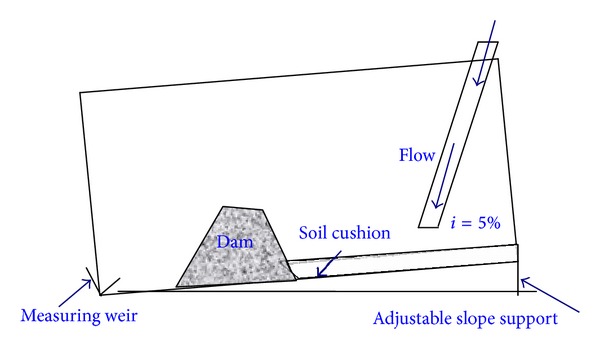
Schematic diagram of the experimental flume.

The dam break experiments are carried out in a rectangular artificial flume 4.5 m long, 1 m high, and 0.4 m wide. The inclination of the flume is fixed at 5%, as shown in Figure 2. 

As shown in Figure 2, a layer of the soil particles is used upstream of landslide dam to better approximate the actual environment. The water flow is controlled with water supply equipment. A measuring weir is positioned at the end of experimental flume to determine the released water flow rates when the dam break occurs.

The majority of the material that makes up the natural landslide dam varies in particle size from 40 mm to 400 mm. To better simulate the real landslide dam, we scaled down the experimental material particle sizes by a factor of 20.  Table 1 shows the particle size distribution of the natural and experimental dams.

**Figure 3:**
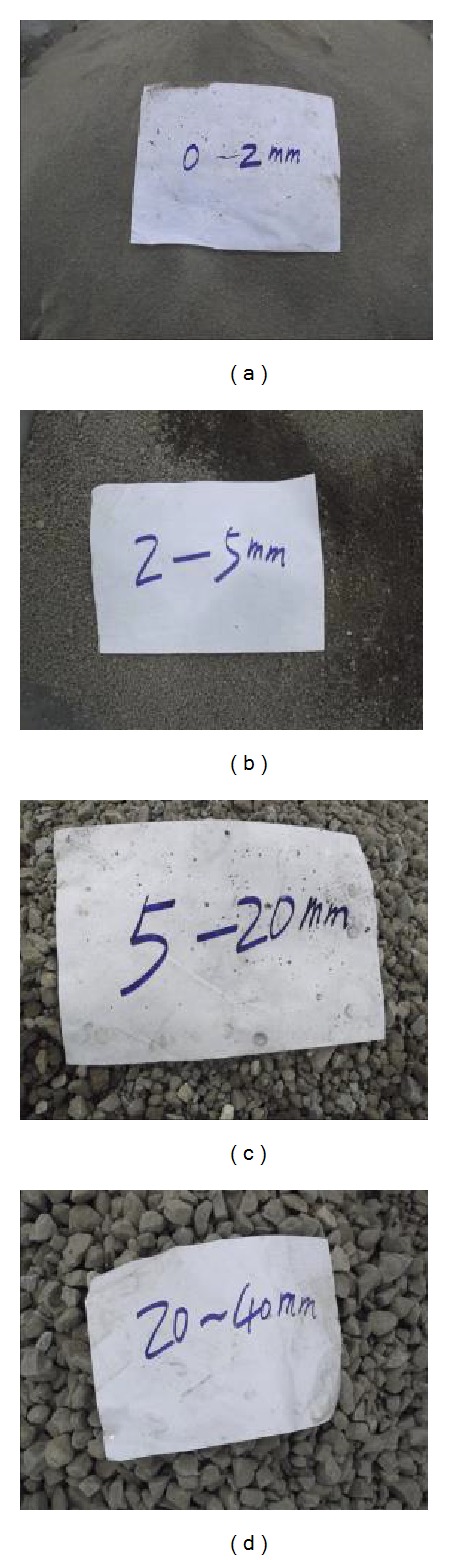
Rock and soil particles used for the experiments, (a) 0–2 mm, (b) 2–5 mm, (c) 5–20 mm, and (d) 20–40 mm.

The experimental dam materials are the mixture of limestone fragments, clay, and silty clay. The particle size distribution of the experimental dam is as the same as for the natural dam scaled down by a factor of 20.  Figure 3 shows the rock and soil particles used in the experiments.

**Figure 4:**
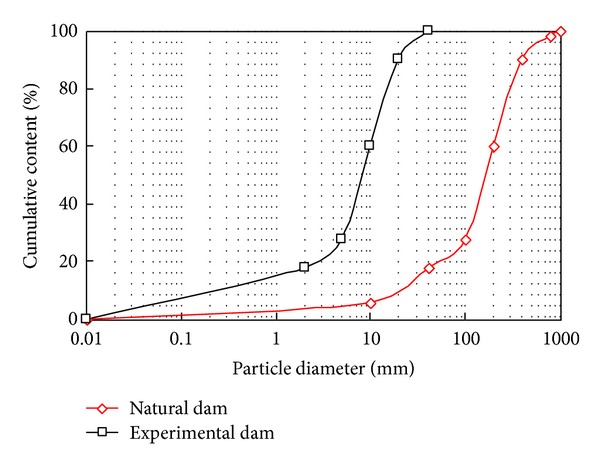
Particle size distribution comparison of the natural dam and the experimental dam.

As shown in Figure 3, the rock and soil particles were taken from the natural landslide dam, air-dried, and sorted by size. During the laboratory tests, the rock and soil particles are remixed to construct the landslide dam.  Figure 4 shows the particle size distribution comparison of the natural dam and the experimental dam. 

### 2.3. Experimental Design

**Figure 5:**
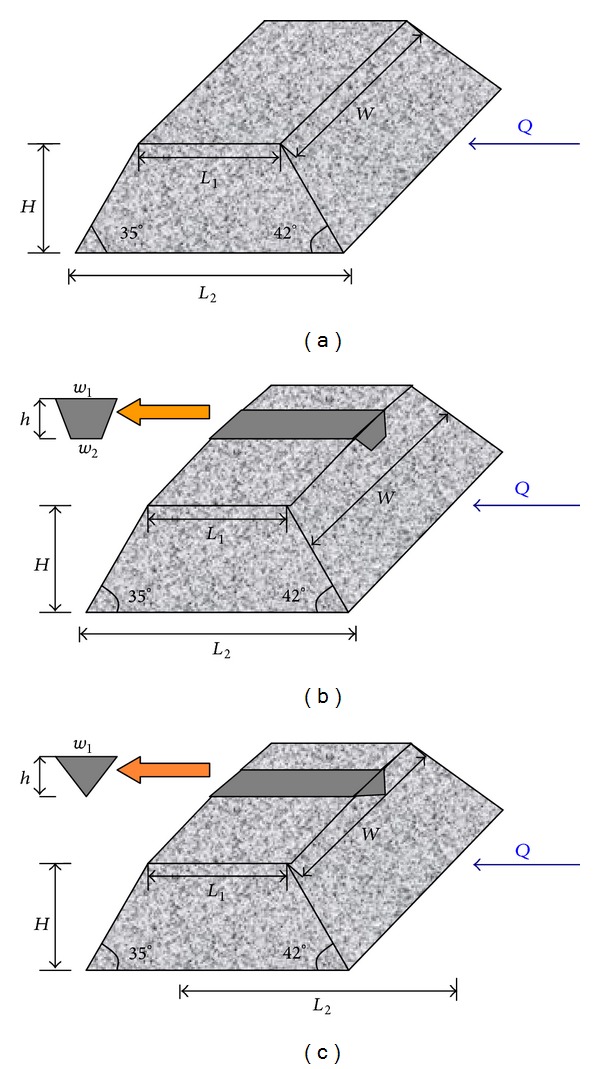
Schematic diagram of the different types of landslide dams used in this experiment: (a) basic landslide dam, (b) landslide dam with a trapezoidal discharge channel, and (c) landslide dam with a triangular discharge channel.

Three types of landslide dams were used in the experiments: a basic landslide dam without any discharge channel, a landslide dam with a trapezoidal discharge channel, and a landslide dam with a triangular discharge channel (as shown in Figure 5).

As shown in Figure 5, *L*_1_ is the top length of the landslide dam; *L*_2_ is the bottom length of the landslide dam; *W* is the width of landslide dam; *H* is the height of landslide dam; *w*_1_ is the top width of the discharge channel; *w*_2_ is the bottom width of the discharge channel; and *h* is the depth of the discharge channel. 

To perform the experiments, the size of landslide dam needs to be scaled down.  Table 2 shows the scaling ratio between the natural dam and experimental dam.

As shown in Table 2, two scaling ratios (400, 800 and 400, 540) are used for the top and bottom lengths of landslide dam, respectively. The scaling ratio is 400 for the width and height of landslide dam. Two scaling ratios (175, 100) are used for the depth of the discharge channel, and one scaling ratio (100) is used for the top and bottom widths of the discharge channel. Previously published studies indicate that average discharge of the Mianyuan river is *≈*10 m^3^/s with a peak flow of *≈*200 m^3^/s. This study uses three different water flow rates (*Q*); 0.1 L/s, 0.2 L/s, and 2.0 L/s.

The slope of the landslide dam is 42° on the upstream side and 35° on the downstream side; these values are held constant for the entire experimental dam. The landslide dam was analysed using 9 dam break mechanism experiments. Four factors that impact dam breaks of landslide dams are considered during in the experiments: (a) water flow conditions, (b) the effects of boulders at the top of the landslide dam, (c) dam size, and (d) discharge channel characteristics.  Table 3 shows the experimental arrangement for the dam break analysis. 

**Figure 6:**
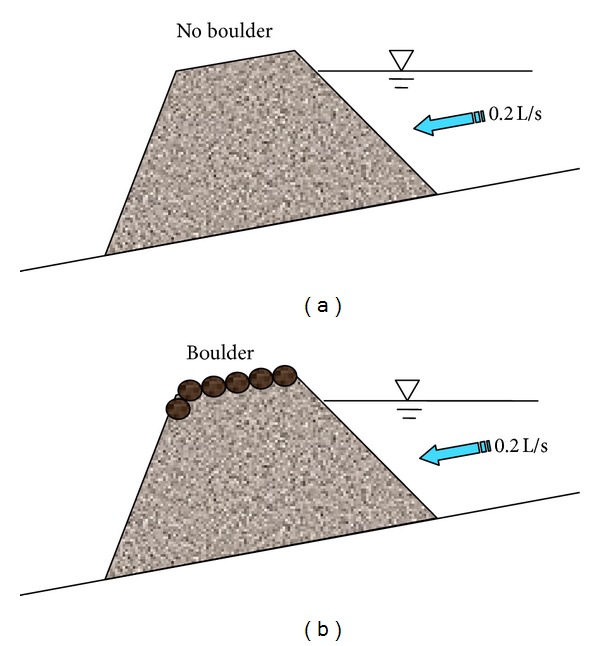
Experimental schemes for the impact of boulder at the topper dam.

As shown in Table 3, three different water flow conditions (0.1 L/s, 0.2 L/s, and 2.0 L/s) are used for each landslide dam, both with and without discharge channels. The top length, bottom length, width, and height of the dams are 26 cm, 50 cm, 40 cm, and 15 cm, respectively. To study the impact of boulders on the dams, boulders are arranged at the top of the landslide dam (experimental set 4) when the water flow is 0.2 L/s (Figure 6). To investigate the influence of dam size, a smaller landslide dam is tested with a water flow of 0.2 L/s (experimental set 5). Two different discharge channel shapes are excavated in the middle of the top of the landslide dams: trapezoidal and triangular. Additionally, we analysed different water flow rates and dam sizes to investigate how dam break failure mechanisms are influenced by the discharge channel (experimental sets 6, 7, 8, and 9). 

The water flow is controlled by a small pump and kept constant during the experiments. A triangle glass measuring-weir at the end of the glass channel measures the dam break flow. Two video cameras are used to monitor the entire experimental process at the front and side of the flume. 

## 3. Results and Discussions

### 3.1. Impact of Water Flow Condition

**Figure 7:**
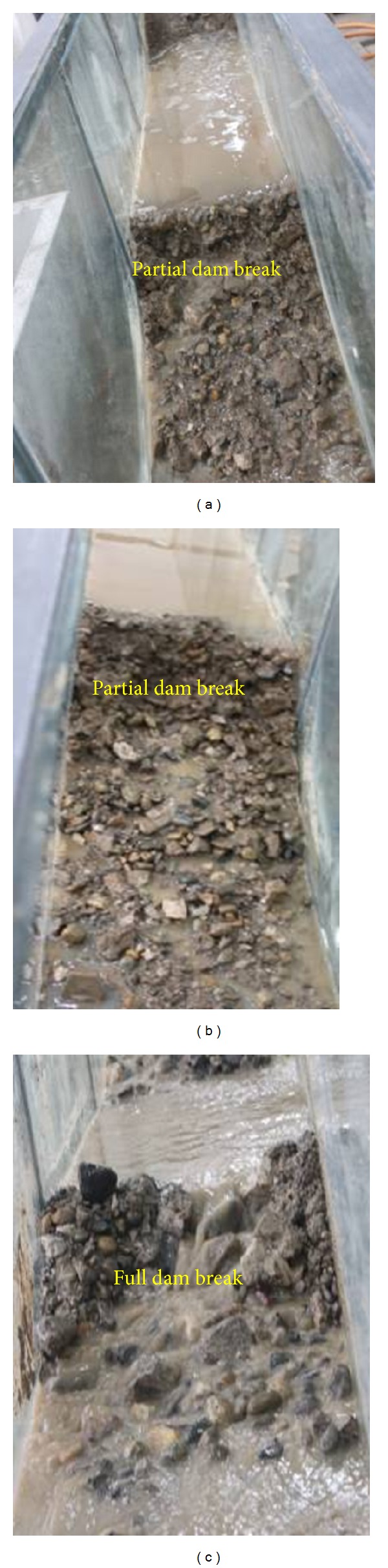
Final patterns of dam break influenced by the water flow conditions: (a) water flow is 0.1 L/s; (b) water flow is 0.2 L/s, and (c) water flow is 2.0 L/s.

The conditions of water flow play a key role in the dam break of the landslide dam. At the initial stages the water flow is blocked by the landslide dam resulting in an increase in water level behind the dam. With a continually increasing water level, overflow occurs at the top of the landslide dam, putting it in an erosional state. Because the landslide dam is loosely consolidated and highly permeable, erosional failure will easily occur.  Figure 7 shows the final pattern of dam break influenced by different water flow conditions. 

**Figure 8:**
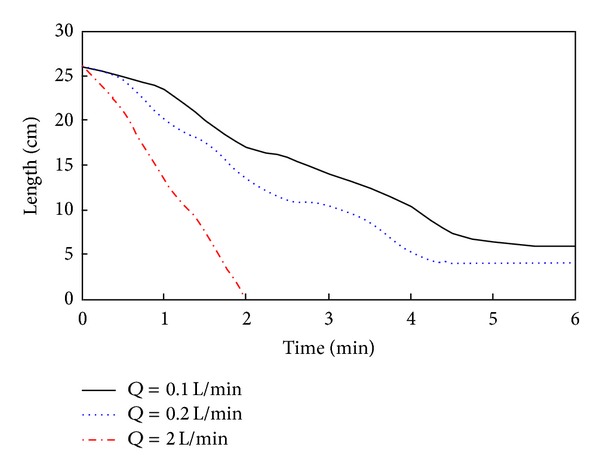
Evolution of the length of the top of landslide dams under different water flow conditions.

As shown in Figures 7(a) and 7(b), when the water flow rate is relatively low, the mean flow velocity is corresponding low, and the erosional force on the landslide dam is small. The landslide dam is stable as long as overflow has not occurred; after overflow begins, the landslide dam fails by toppling. Progressive failure occurs downstream of the landslide dam when the water flow is 0.1 L/s and 0.2 L/s. As shown in Figure 7(c), large water flow results in a large erosional force on the landslide dam. A dyke breach forms in the middle of the landslide dam, and the landslide dam fails rapidly. Furthermore, at the end of the experiment, only part of the downstream dam is broken when the water flow rates are 0.1 L/s and 0.2 L/s. However, when the water flow rate is 2.0 L/s, the entire dam breaks.  Figure 8 shows the evolution of the length of the top of the landslide dams under different water flow conditions (*time* = 0* is the time of initial dam failure for the evolution process curves*). 

**Figure 9:**
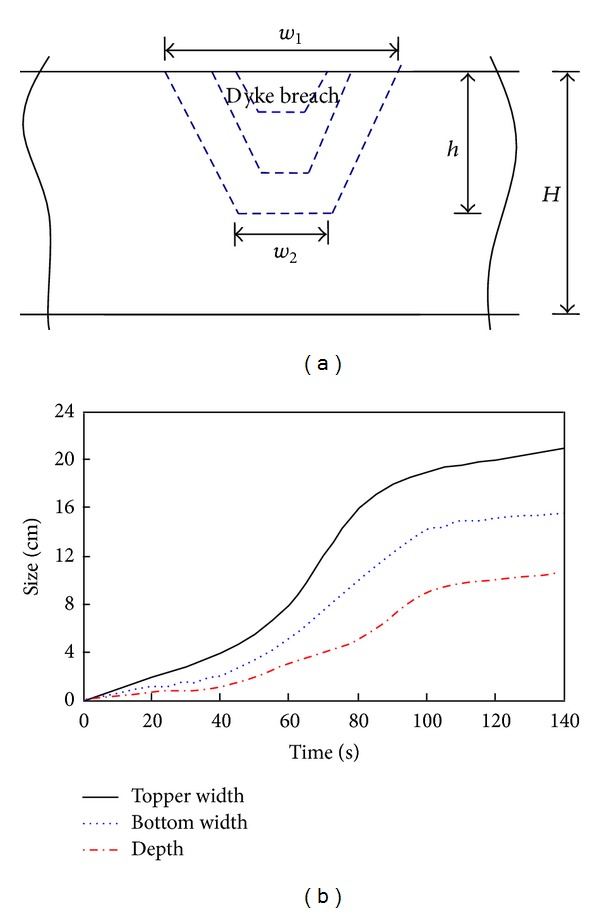
Evolution of the dyke breach when the water flow is 2.0 L/s: (a) schematic diagram and (b) evolution of the size of dyke breach.

As shown in Figure 8, different water flow conditions result in different rates of erosion of the landslide dam, and the failure speed of a landslide dam increases with increasing water flow rates. When water flow rates are 0.1 L/s or 0.2 L/s the strength of the dam materials decreases rapidly when water crosses the crest of the dam, and small particles are washed away by the water flow. Small collapses occur on the top of the downstream side of the dam with progressive collapses occurring faster and more rapidly. When the dam failure reaches a certain point, the landslide dam can maintain relative stability because of the large particles that have not been washed away. When the water flow achieves an equilibrium state, the upstream flow is approximately balanced with the discharge, and dam failure ceases. Compared, dam break patterns for water flow rates of 0.1 L/s and 0.2 L/s, larger flow rates result in a rapid failure of landslide dams. If the water flow is large enough, the erosional force of the water is enormous and the failure speed of landslide dam is very rapid. In this scenario, the entire landslide dam collapses causing an enormous flood downstream of the dam. When the water flow is large enough (2.0 L/s), a dyke breaches the middle of the landslide dam and is gradually expanded by the flowing water. Ultimately, the rapid water flow results in total dam failure.  Figure 9 shows the evolution of the dyke breach when the water flow rate is 2.0 L/s. 

In Figure 9(a), the depth of dyke breach is *h*, the top width of dyke breach is *w*_1_, and the bottom width of the dyke breach is *w*_2_. After the first failure of the landslide dam, the size of the dyke breach (including top width, bottom width, and depth) increases due to continuous water flow. In the early stages, overflow results in a small dyke breach, but the size of dyke breach increases slowly. The collapse speed accelerates until the dam achieves stability (Figure 9(b)). When the flow rate is large, the width of the dyke breach increases quickly. 

### 3.2. Impact of Boulders on the Top of the Dam

**Figure 10:**
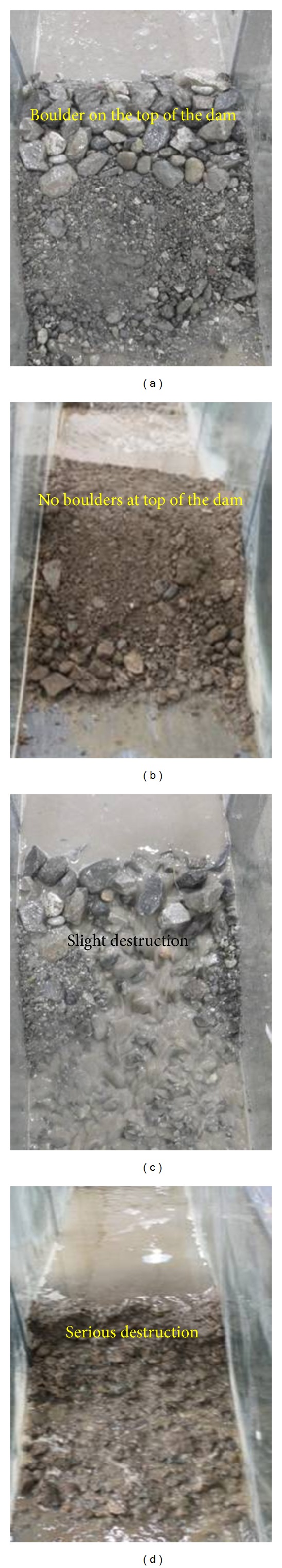
Experiments on the impact of boulders on the top of the dam on dam break mechanisms: (a) landslide dam with a boulder-covered top before dam break, (b) landslide dam without a boulder-covered before dam break, (c) after dam break of a landslide dam with a boulder-covered top (d) after dam break of a landslide dam without a boulder-covered.

During overflow, the resistance to erosion of the landslide dam depends on the size of dam materials and the velocity of water flow. Fine particles are easily transported downstream by water flow, whereas coarse particles are not. The resistance to erosion of boulders is larger than for fine particles, so that, for all of the landslide dams triggered by the Wenchuan earthquake, those that are composed mainly of boulders can be preserved over several rainy seasons. Most of the landslide dams made mainly of soil, however, have disappeared. The resistance to erosion of a dam's material directly impacts the mechanisms of dam break of a landslide dam. Here, we design two comparable experiments to analyse how the mechanisms of dam break are influenced by boulders at the top of the dam (Figures 10(a) and 10(b)). These two dams are the same size, experience the same water flow conditions, and are composed of the same material. The only difference between these two dams is that one has boulders on the top of the dam and another one does not. 

As shown in Figure 10(c), during overflow, even though water penetrated the dam, and some local failures have occurred downstream of the landslide dam, dam break has not occurred because of the significant erosional resistance of the boulders on the top of the dam. As shown in Figure 10(d), during overflow, the shear strength of the dam's material decreases and the landslide dam fails; fine particles and small rock blocks are carried away downstream by the flowing water. Compared with Figure 10(c), Figure 10(d) illustrates that boulders on the top of the dam can control erosion of the landslide dam when the water flow rate is under a certain value. However, if the velocity of the water is high enough, then the landslide dam will break. Boulders can be added to the top of a dam to control erosion by water and increase the self-weight of the dam, resulting in the more stability of the downstream side of a landslide dam. 

### 3.3. Impact of Dam Size

**Figure 11:**
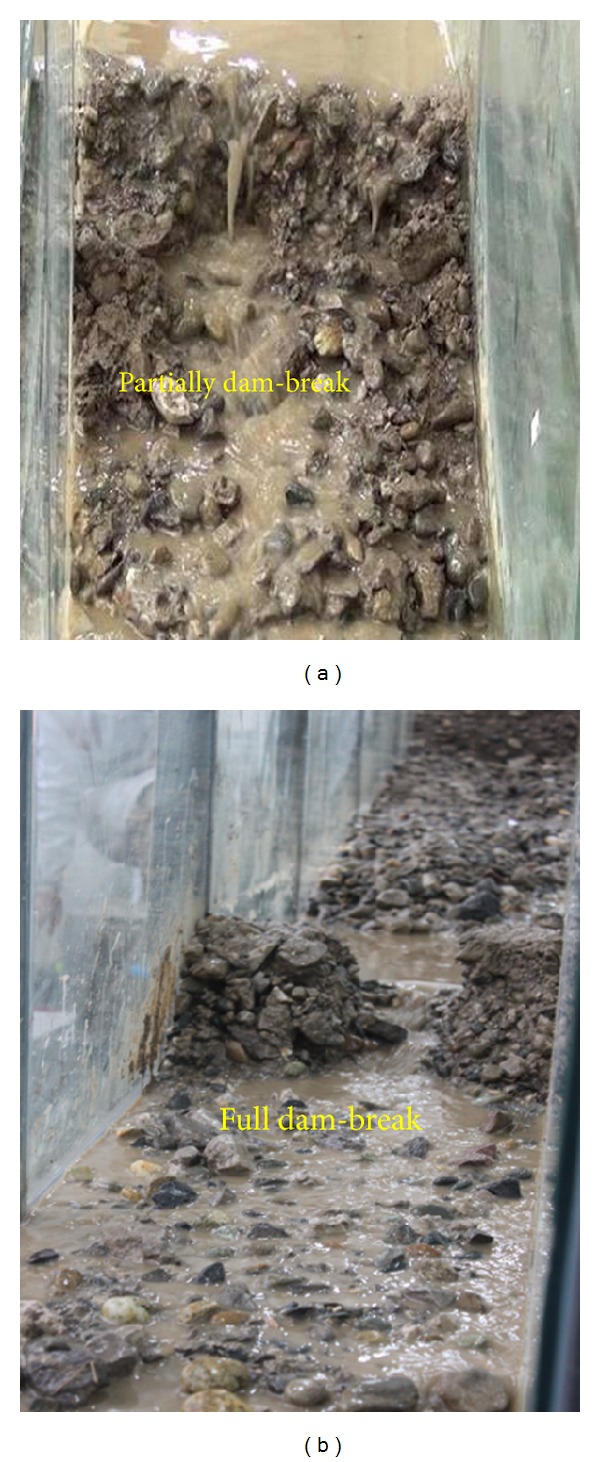
The results of Dam break experiments influenced by dam size: (a) top length of 26 cm and (b) top length of 13 cm.

Two different dam-top lengths were used to analyse the impact of dam size impact on dam break mechanisms. The resulting patterns of the dam exhibit different characteristics based on the initial size of the dam.  Figure 11 shows the results of dam break for different dam sizes. 

**Figure 12:**
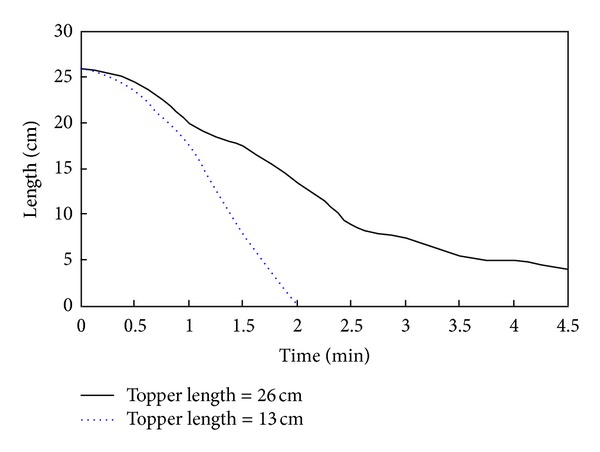
Evolution of the top length of a landslide dam for different sized dams.

As shown in Figure 11, the length of the dam has a significant influence on dam break processes. When the top length of a dam is 26 cm, only partial dam break occurs. However, when the topper length of dam is 13 cm, full dam break occurs rapidly, resulting in a large peak flow of discharge. A dyke eventually breaches in the middle of the landslide dam. If the dam is thinner, the stability and resistance to erosion are relatively low, and dam break occurs easily under normal water flow conditions.  Figure 12 shows the evolution of the top length of landslide dams for different dam sizes. 

**Figure 13:**
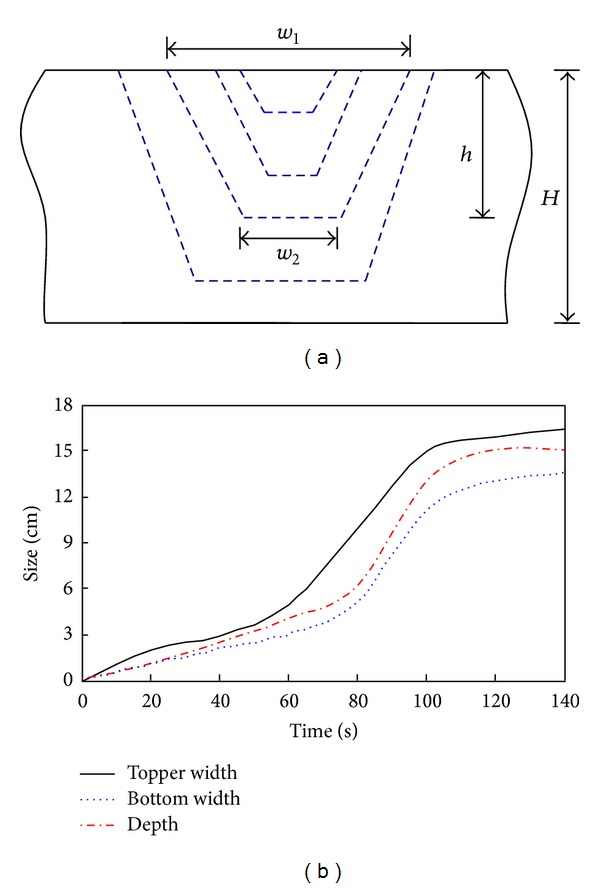
Evolution of the dyke breach when the top length of the dam is 13 cm: (a) schematic diagram and (b) evolution of the size of the dyke breach.

As shown in Figure 12, the smaller the length of the dam the shorter the time of dam break, and the larger the degree of destruction. When the length of the dam is small enough, the dam is like a beam that cannot support large water pressures and will consequently burst in the middle.  Figure 13 shows the evolution of the dyke breach when the top length of dam is 13 cm. 

As shown in Figure 13, because the dam is relatively narrow it has more vulnerable points. When water overflows the top of the dam, the dam cannot resist the water pressure and erosion. The dam crest will then fail, and as the water penetrates into the dam, the structure of the dam breaks down. Due to the action of the water flow, the size of dyke breach increases rapidly and causes a complete dam break of the landslide dam (Figure 13(b)). From an engineering perspective, if the aspect ratio of landslide dam is very large, then the landslide dam is in a more dangerous state than when the aspect ratio is relatively small. In the area affected by the Wenchuan earthquake, most of the landslide dams with large aspect ratios were broken in the subsequent rainy season; these dams therefore require immediate attention. 

### 3.4. Impact of Discharge Channel

**Figure 14:**
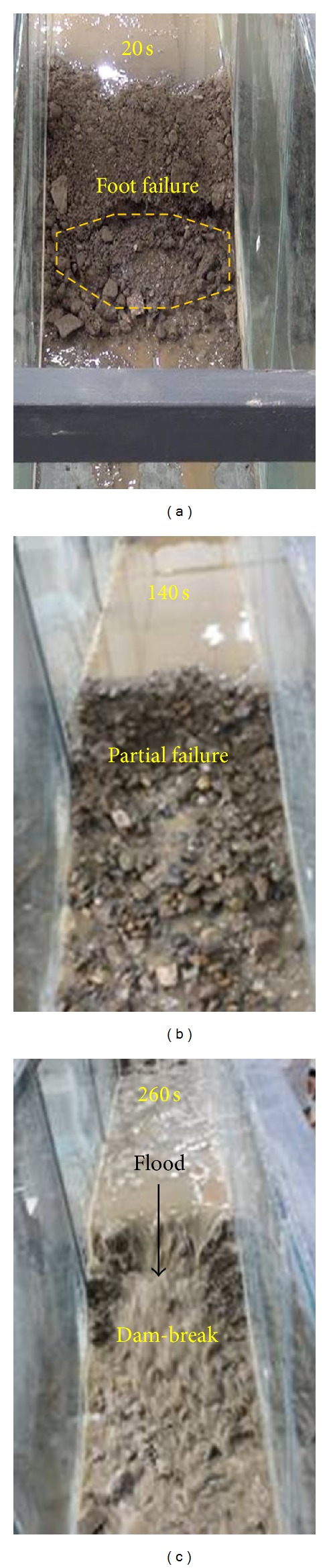
Process of dam break of a landslide dam without any discharge channel at different time intervals: (a) 20 s, (b) 140 s, and (c) 260 s.

The water capacity behind the landslide dam is typically very large and therefore poses a major threat for areas downstream. Flood discharge treatments are always conducted on landslide dams. There are two main types of discharge treatments for landslide dams: a discharge channel and a discharge tunnel. Due to the relatively high cost of a discharge tunnel, the discharge channel is always used to treat landslide dams in emergency situations. After discharge channel excavated the water capacity behind the landslide dam can be controlled. However, the velocity of the water flow in the discharge channel will increase and cause erosion of the landslide dam and may lead to a dam break of landslide dam. The impact of a discharge channel on the dam break process is very important for formulating disaster prevention and mitigation measures. In this experiment, two different shaped discharge channels are excavated at the middle of the landslide dam: trapezoidal and triangular.  Figure 14 shows the process of dam break of a landslide dam without any discharge channel. 

As shown in Figure 14, as the water level increases behind the landslide dam, local failure occurs at the foot of the downstream slope due to increasing pore water pressure and decreasing shear strength of dam's materials (Figure 14(a)). Overflow occurs when water levels increase behind the dam causing water to penetrate the dam and cause progressive failure of the downstream slope of the dam (Figure 14(b)). With continuous failure of the landslide dam, the dam break occurs and causes an outburst flood downstream (Figure 14(c)). 

**Figure 15:**
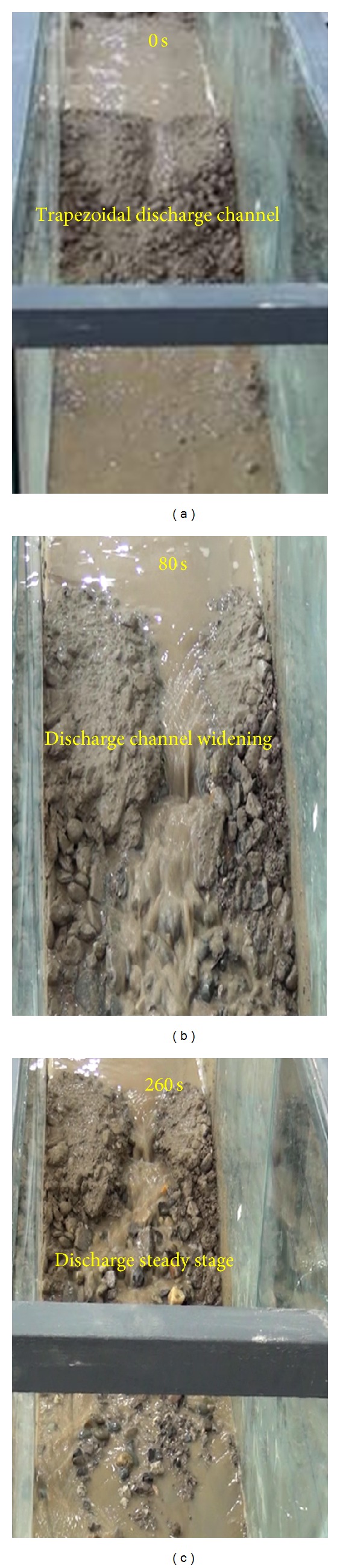
The process of dam break of a landslide dam with a trapezoidal discharge channel at different time intervals: (a) 0 s, (b) 80 s, and (c) 260 s.

The failure process of a landslide dam with a discharge channel differs from a landslide dam without any discharge channel.  Figure 15 shows the process of dam break with a trapezoidal discharge channel. 

As shown in Figure 15(a), in the initial stages of the experiment the water level behind the landslide dam increases with the continued water flow. Flow then initiates through the discharge channel. Flow speed increases due to the reduction in the area of discharge channel, and progressive failures occur on the both sides of the discharge channel. The bottom of the discharge channel is eroded by the water flow, and failures initiate on the downstream slope of landslide dam (Figure 15(b)). Lastly, the water flow reaches equilibrium where upstream flow is approximately balanced by the water discharge, and dam failures cease (Figure 15(c)). 

**Figure 16:**
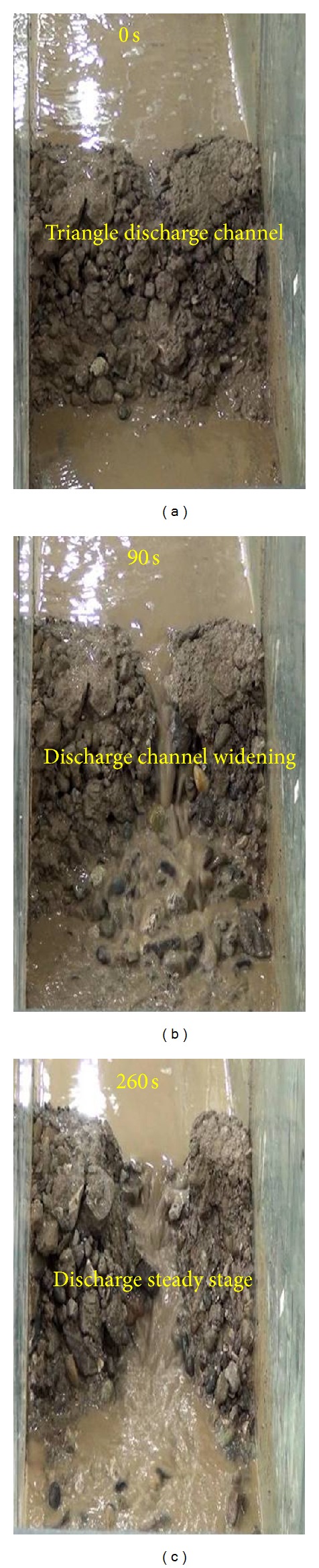
Process of dam break of a landslide dam with a triangular shaped discharge channel at different time intervals: (a) 0 s (b) 90 s and (c) 260 s.

A landslide dam with a triangle discharge channel is used to study the effects of different discharge channel shapes on the mechanisms of dam break.  Figure 16 shows the process of dam break of a landslide dam with a triangular shaped discharge channel. 

As shown in Figure 16, the failure of a landslide dam with a triangular shaped discharge channel is largely similar to that for a trapezoidal discharge channel. However, the depth and speed of erosion in the triangular discharge channel are larger than for a trapezoidal discharge channel because the area of the discharge channel is smaller, resulting in a higher flow velocity. The erosional depth and width of the triangular discharge channel are both larger than for a trapezoidal discharge channel, and progressive failures also occur on the downstream slope of landslide dam. The failure volume of dam for landslide dam with triangle discharge channel is larger than the dam with trapezoidal discharge channel. A triangular discharge channel is therefore not a reasonable emergency treatment for a landslide dam. 

**Figure 17:**
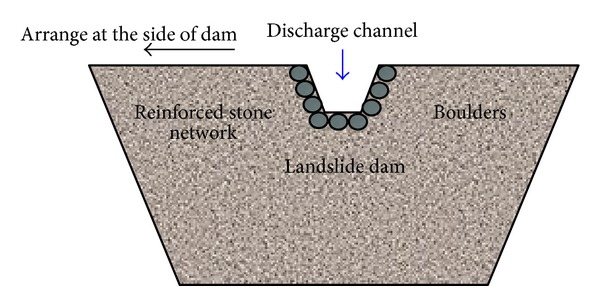
Suggestion for the design of a discharge channel for the emergency treatment of a landslide dam.

Trapezoidal discharge channels are suitable flood discharge measures for the landslide dams.  Figure 17 shows a suggestion for the design of a discharge channel for the emergency treatment of a landslide dam. 

As shown in Figure 17, the discharge area of a channel should satisfy spillway requirements, and the discharge channel should be located on the side of the landslide dam, not in the middle, In addition, reinforcement measures, such as boulders or reinforced stone networks, should be used to protect both sides and the bottom of discharge channel. 

**Figure 18:**
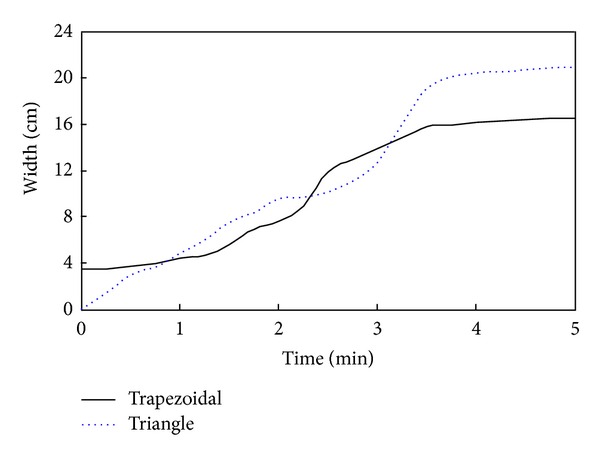
Evolution for the bottom width of different shapes of discharge channels.

A discharge channel can control the water level behind the landslide dam. However, flow velocity will increase and cause erosion of both of the sides and the bottom of the discharge channel. The stability of a landslide dam with a discharge channel can decrease and result in a full dam break that causes catastrophic disasters.  Figure 18 shows the evolution for the bottom width of different shapes of discharge channels. 

As shown in Figure 18, for a landslide dam with a trapezoidal discharge channel, after water begins to flow across the discharge channel the width of the channel initially increases slowly. When the discharge approaches peak flow, the size of discharge channel increases rapidly. Ultimately, the size of the discharge channel stops growing, indicating that the water flow has reached equilibrium. For a landslide dam with a trapezoidal discharge channel, after water begins to flow across the discharge channel, the width of the discharge channel continues to increase with increasing water flow and can transition into a state flow stage; however the final width of the discharge channel is larger than for the trapezoidal discharge channel. 

## 4. Dam Break Mechanism of the Landslide Dam

In this section, following a comprehensive analysis of both experimental data and field investigations, the dam break mechanisms of landslide dams are presented and compared to the dam break of the Jiadanwan landslide dam. 

### 4.1. Dam Break Mechanism Analysis

**Figure 19:**
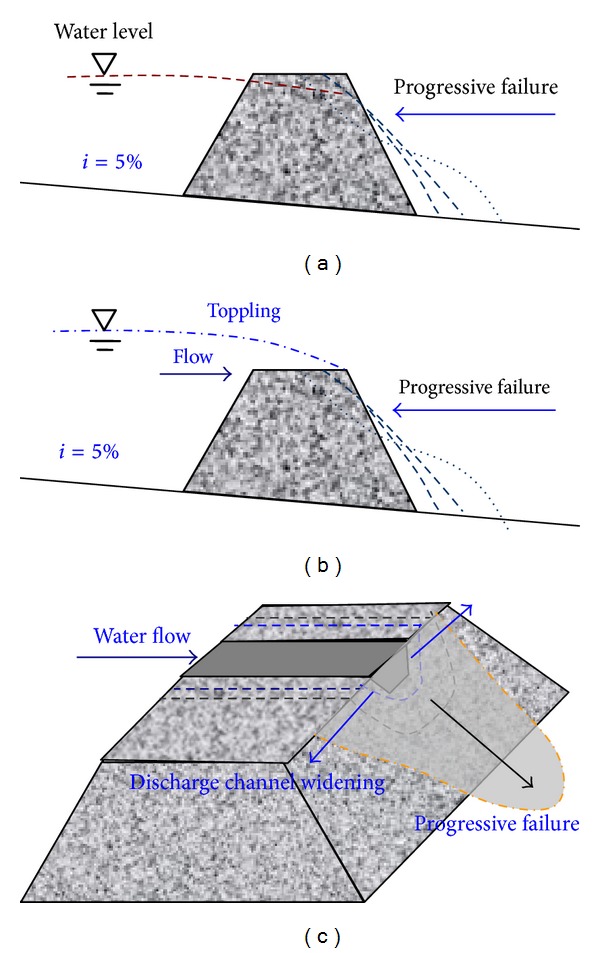
Three dam break models for landslide dams: (a) progressive penetration failure, (b) progressive failure and erosion by the toppling flow, and (c) erosion failure for the discharge channel.

Previous research has suggested that dam breaks are transient bursts, and many peak flow-forecasting formulas use this assumption. However, in certain situations, the failure of a landslide dam is a progressive process. The dam break mechanism of a landslide dam depends on many factors, including material composition, mechanical properties, flow conditions, and dam shape. Here, three typical dam break models for a landslide dam are presented (as shown in Figure 19).

As shown in Figure 19(a), progressive penetration failure always occurs when the shear strength of the dam's materials is relatively low. The increasing pore water pressure in the dam and the decreasing shear strength of the dam's materials results in landslide dam failure and causes disastrous floods downstream. As shown in Figure 19(b), this dam break model occurs when the landslide dam is relatively stable; however, the erosional resistance of the dam's material is not great enough to experience flow erosion on the top of the dam. Progressive failure of landslide dams will occur and cause enormous and disastrous floods. As shown in Figure 19(c), when the water flows through the discharge channel, the velocity of water increases due to the reduction in the spillway area. If the dam's materials on both the sides and the bottom are not resistant to erosion, failures occur, causing a widening of the discharge channel. Progressive failures occur on the downstream slope of the landslide dam. 

### 4.2. Comparison of Experimental Data and Field Test Results

**Figure 20:**
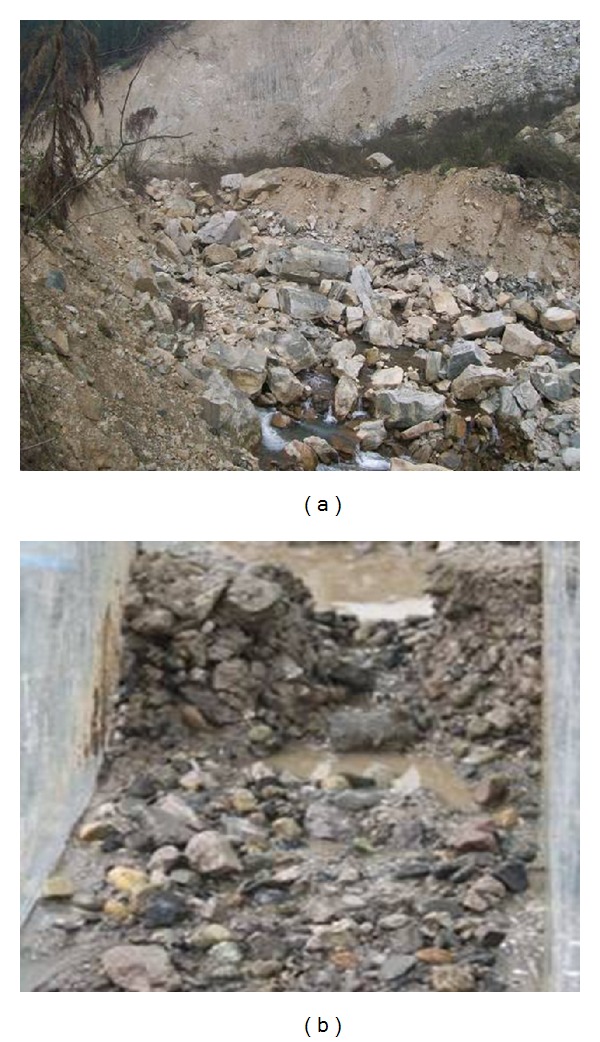
Dam break patterns for the landslide dam: (a) actual situation and (b) experimental result.

After the formation of the Jiadanwan landslide dam, a discharge channel was excavated on the left bank of the landslide dam in an attempt to reduce the threat of dam break and downstream flooding. The discharge channel has a bottom width of 6 m, a depth of 7 m, a length of 540 m, and has sides that both slope at 30°. However, during the following rainfall season, the dam partially broke due to the discharge channel, resulting in an increase in the downstream river bed height of 2-3 m (as shown in Figure 20(a)).  Figure 20(b) shows the experimental results for the dam break pattern of the Jiadanwan landslide dam. 

As shown in Figure 20(b), the actual dam break is similar to the experimental dam break. After the dam breaks, tiny particles are transported away and the larger particles collapse. Because the channel slope is low, a large number of particles are trapped downstream close to the dam, with the larger particles left on the surface. The experiment and the field site observations are generally consistent, which agree with the general models of dam breaks. 

## 5. Conclusions

Dam breaks of landslide dams are disastrous to property and life downstream. Fully understanding the process of dam break is very important for reducing damage and formulating effective disaster prevention and mitigation measures. This study uses the Jiadanwan landslide dam as a classic example of a modern landslide dam. Laboratory tests are used to study the dam break mechanisms of landslide dams. Four factors considered in these experiments that impact the dam break of a landslide dam are the following: (a) water flow conditions, (b) the effects of boulders on the top of a landslide dam, (c) dam size, and (d) discharge channel characteristics. 

Experimental results show that partial dam break occurs downstream of the landslide dam when the water flow is 0.1 L/s and 0.2 L/s, and full dam break of the landslide dam occurs when the water flow is 2.0 L/s. Boulders at the top of a dam can minimise erosion of a landslide dam when the water flow is under a certain value. If the dam is thinner, the stability and erosion resistibility is relatively poor, and dam break occurs easily under normal water flow conditions. The stability of a landslide dam with a discharge channel can be decreased and result in a full dam break. 

Experiments are an effective method to investigate the mechanisms of dam break of landslide dams. However, experiments are limited by model size. The scaling relationships of models and natural landslide dams can be complicated and result in significant differences between experiments and field observations.

## Figures and Tables

**Table 1:** Particle size distribution of the natural dam and experimental dam.

Natural dam (mm)	Experimental dam (mm)	Cumulative content (%)
0–40	0–2	18
40–100	2–5	28
100–200	5–10	60
200–400	10–20	90
400–800	20–40	100

**Table 2:** Scaling ratio between the natural dam and experimental dam.

Parameter	Symbol (unit)	Nature	Experimental	Scaling ratio
Landslide dam				
Topper length	*L*_1_ (m)	105	0.26, 0.13	400, 800
Bottom length	*L*_2_ (m)	200	0.50, 0.37	400, 540
Width	*W* (m)	160	0.40	400
Height	*H* (m)	60	0.15	400
Discharge channel				
Topper width	*w*_1_ (m)	7	0.07	100
Bottom width	*w*_2_ (m)	3	0.03	100
Depth	*h* (m)	3.5	0.020, 0.035	175, 100

**Table 3:** Experimental setup for the dam-break analysis of landslide dam.

Set	Landslide dam size				Discharge channel				Boulder at top of landslide dam	*Q* (L/s)
	*L*_1_ (cm)	*L*_2_ (cm)	*W* (cm)	*H* (cm)	Shape	Size/cm				
						*w*_1_	*w*_2_	*h*		
1	26	50	40	15	—	—	—	—	N	0.1
2	26	50	40	15	—	—	—	—	N	0.2
3	26	50	40	15	—	—	—	—	N	2
4	26	50	40	15	—	—	—	—	Y	0.2
5	13	37	40	15	—	—	—	—	N	0.2
6	26	50	40	15	Trapezoid	7	3	3.5	N	0.1
7	26	50	40	15	Trapezoid	7	3	3.5	N	0.2
8	26	50	40	15	Trapezoid	7	3	2.0	N	0.2
9	26	50	40	15	Triangle	7	0	3.5	N	0.2
